# T3 levels in relation to prognostic factors in breast cancer: a population-based prospective cohort study

**DOI:** 10.1186/1471-2407-14-536

**Published:** 2014-07-24

**Authors:** Ada Tosovic, Anne-Greth Bondeson, Lennart Bondeson, Ulla-Britt Ericsson, Jonas Manjer

**Affiliations:** Department of Surgery, Skåne University Hospital Malmö, Lund University, Malmö, Sweden; Department of Pathology, University and Regional Laboratories Region Skåne, Lund University, Malmö, Sweden; Department of Endocrinology, Skåne University Hospital Malmö, Lund University, Malmö, Sweden; Department of Reconstructive Surgery, Skåne University Hospital Malmö, Lund University, Malmö, Sweden

**Keywords:** Breast cancer, Triiodothyronine, Prognostic factors

## Abstract

**Background:**

The issue of a potential association between thyroid conditions/hormones and breast cancer has been studied extensively during the last decades but the results have been inconclusive and almost no studies have investigated breast cancer aggressiveness. We have previously found a positive association between prospectively measured levels of triiodothyronine (T3) and breast cancer incidence as well as breast cancer mortality. We now investigated prediagnostic T3 levels in relation to specific prognostic factors in breast cancer.

**Methods:**

The Malmö Preventive Project is a population-based prospective cohort including 2185 women in whom T3 levels were measured at baseline. That is, total T3 levels were measured before a potential diagnosis of breast cancer. Mean follow-up was 23.3 years and 149 women in the study population were diagnosed with invasive breast cancer. Tumours were classified according to selected prognostic factors of breast cancer; i.e. grade, tumour size, lymph node metastasis, and hormonal receptor status. T3 was handled both as tertiles and as a continuous variable. A Cox’s proportional hazards analysis yielded hazard ratios with 95% confidence intervals. All analyses were also restricted to postmenopausal women.

**Results:**

Overall there was a statistically significant association between T3 and “all” breast cancers. The adjusted Hazard Ratio (HR) in the third tertile, as compared to the first, was (1.61:1.07-2.43). There was a statistically significant positive association between the third T3 tertile and large tumours, i.e. > 20 mm, (3.17:1.20-8.36) and the occurrence of lymph node metastases, (4.53:1.60-12.83). Other prognostic factors positively associated with T3 were negative oestrogen receptor (ER) status, (3.52:1.32-9.41) and negative progesterone receptor (PGR) status, (3.52:1.42-8.75). The analyses of T3 as a continuous variable and analysis restricted to postmenopausal women, confirmed the results but also showed an association with smaller tumours and in postmenopausal women a contemporary association with negative lymph nodes.

**Conclusions:**

This prospective study of serum T3 levels in relation to breast cancer aggressiveness is the first of its kind. We found statistically significant positive associations between higher prediagnostic T3 levels and larger tumours, occurrence of lymph node metastases, and negative ER and PGR status.

## Background

Thyroid disorders and breast cancer are both common in postmenopausal women, and an association between these conditions would have a large clinical impact. The issue of a potential association between thyroid conditions/hormones and breast cancer risk has been studied extensively during the last decades but the results have so far been inconclusive [1–3]. Interpretation of previous studies is difficult as almost all have been cross-sectional. This is a major problem when measuring thyroid hormones in breast cancer patients as the disease *per se*, as well as its treatment, may affect hormonal levels.

In order to investigate whether there is an association between thyroid hormones and breast cancer, the most relevant design would be to use pre-diagnostically measured hormonal levels.

The overwhelming part of previous studies on thyroid hormones and breast cancer, has investigated the risk of breast cancer. Another aspect is clinical outcome, e.g. survival. To our knowledge, only two previous studies, including 84 and 47 patients respectively, have investigated thyroid hormones in relation to survival following breast cancer, but they found no clear association [4, 5]. The literature on thyroid disorders and breast cancer aggressiveness or prognostic factors is also very scarce. One study has shown that thyroid autoimmunity in relation to breast cancer aggressiveness is associated with a lower frequency of distant metastasis [6]. Another cross-sectional study on thyroid conditions and breast cancer aggressiveness found no relation to the histopathological grade but a higher frequency of metastatic lymph nodes and vascular invasion in patients with thyroid pathology [7]. However, an early cross-sectional study by Lemaire et al. showed no relation between thyroid function and TNM stage of breast cancer [8].

In two recent prospective studies, we found a positive association between prediagnostic thyroid hormone levels and the risk of developing breast cancer [9, 10]. Furthermore, we have also – based on the same cohort as one of these studies – shown a positive association between prediagnostic triiodothyronine (T3) levels and breast cancer mortality [11]. Total T3 and not T4 was chosen as the exposure in that and the present study, since at baseline, when blood samples for thyroid status were collected, only total T3 and TSH were analysed.

A question that remains to be answered is if this high mortality is related to a higher incidence or more aggressive forms of breast cancer.

The aim of the present study was to investigate the association between prediagnostic T3 levels and prognostic factors related to breast cancer aggressiveness; i.e. tumour size, grade, lymph node metastases, and hormonal receptor status.This was studied in a prospective cohort of 2185 women, out of whom 149 were diagnosed with invasive breast cancer during a follow-up of at least 18 years.

## Methods

### The malmö preventive project

Originally, 10 902 women participated in the Malmö Preventive Project. The project was established in 1974 when residents in Malmö, a city in southern Sweden, were invited to participate in a health survey. Entire birth cohorts, men and women, were examined until 1992 when the department closed. Approximately 70% of invited subjects participated [12].

All women answered a questionnaire concerning sociodemographic information, lifestyle habits, and medical history. Questions on reproductive factors, i.e. oral contraceptives (OC) and menopausal status, were included in women screened from April 1983 and onward. BMI (kg/m2) was assessed at baseline examination [12]. A subject was considered to have a history of goitre if the question ‘have you been treated for goitre’ was answered with ‘yes’. The baseline questionnaire included no questions on specific type of previous thyroid disorders or previous medications.

The present study was approved by the ethical committee at Lund University: Dnr 652/2005 and Dnr 501/2006.

All former participants were informed about the present study by newspaper as required by the local ethical committee. All participants were offered to be excluded from the present study if they wished so, by contacting the authors via an enclosed telephone number. None of the participants took contact.

### T3 analysis

Blood samples were taken after an overnight fast with the patient in the supine position. The serum samples were analysed at baseline for total T3 and TSH in women born in 1928 and 1941 and examined in 1983 and 1984. In women born in 1935 (examined from 1990 to 1992), total T3 was measured in a subset of all women, i.e. those with pathological TSH values, a history of thyroid disease, or those with an enlarged thyroid gland at examination. In addition to this, the attending physician could also decide to analyse T3 [5]. Total T3 was measured by a double antibody RIA (reference interval 0.9–3.2 mmol/l) [13]. Only six women had a value above the upper reference limit.

### Study cohort and follow-up

Among 10 902 women, reproductive data including menopausal status had been assessed in 8051 subjects. T3 had been measured in 2383 women (1161 born in 1928, 907 born in 1941, and 315 born in 1935). Women with prevalent breast cancer (n = 35), goitre (n = 167), or both (n = 4) were identified and excluded from this analysis. Finally, the study population consisted of 2185 women with information on T3 and without breast cancer at baseline or a record of goitre. Tumour endpoints were retrieved by record linkage with the Swedish Cancer Registry and the Southern Swedish Regional Tumour Registry until the end of follow up, the 31 December 2010. Following baseline examination, 149 women in the study population were diagnosed with invasive breast cancer during a mean follow-up of 23.3 years (standard deviation 6.2) and total follow-up included 50 807 person years. Information on vital status was retrieved from the Swedish Cause-of-Death Registry up until 31 December 2010.

### Tumour grading and staging

The evaluation of tumour samples diagnosed until December 2004, was originally performed as part of a previous study [14]. The tumour samples were re-evaluated by three senior pathologists. Histologic grade was assessed according to the Nottingham classification (NHG) as previously described [15]. Information on tumour characteristics in breast cancers diagnosed from January 2005 until December 2010 was obtained from the original pathology reports. Lymph node status and tumour size, were obtained from pathology reports. Size was divided into two groups with a cut-off at 20 mm, the size that discriminates T1 from T2 tumours in the TNM classification [16].

### Receptor status

Information on oestrogen receptor (ER) and progesterone receptor (PGR) status in tumours diagnosed before the 30^th^ of April 1997 was obtained by re-examination of collected tumour tissue by two senior pathologists using an immunohistochemical (IHC) method [17]. For tumours in the present study diagnosed following this date, information on receptor status was obtained from the original pathology reports as the IHC method had then been implemented into clinical practice. In 18 cases, information on receptor status was missing. These tumour samples were retrieved and examined, using the same method as above, by a senior pathologist (LB). Receptor staining in more than 10% of the tumour cells was regarded as positive.

### Statistical methods

Distribution of established and potential risk factors for breast cancer was investigated in breast cancer cases and the rest of the cohort. Tertile cut-points for T3 were based on the distribution among all women in the study population. Hazard ratios (HR), with 95% confidence intervals (CI), were calculated using Cox’s proportional hazards analysis. The assumption of proportional hazards was met as tested by log-minus log curves.

For all the analysis a *P* value < 0.05 was considered as statistically significant.

Analyses were subsequently adjusted for age at baseline. The limited number of breast cancer cases in each subgroup did not allow inclusion of all covariates in the same model, but in relation to invasive breast cancer, age and one additional factor at a time were included in the same model. In this way, OC affected the HRs most, with an increase of the HR by a value of 0.14, whereas the other covariates lead to barley any change of the HRs (0.00-0.02). Hence, the only adjusted model used for all different sub groups was the one including age at baseline and OC.

All the analyses were repeated with T3 as a continuous variable. T3 was not normally distributed (p < 0.001 using a one sample Kolmogorov–Smirnov test), but to give estimates more readily interpretable, the original T3 values were used to obtain HRs. However, T3 values were also transformed using the natural logarithm in order to confirm whether the associations were statistically significant or not. The analysis with continuous T3 was stratified according to menopausal status. In a sensitivity analysis, all analyses were repeated excluding cases with diagnosis of breast cancer within two years following baseline.

## Results

Invasive cases had less often had children than women in the rest of the cohort. Never smoking, being married, and abstaining alcohol was also more common in invasive cases. All other potential and established risk factors were evenly distributed in invasive cases and the rest of the cohort (Table 1). The distribution of potential and established risk factors according to serum level of T3 are presented in Table 2.

**Table 1:** **Distribution of established and potential risk factors for breast cancer in women with breast cancer and the rest of cohort**

Factor	CIS n = 21	Invasive n = 149	Rest of cohort* n = 2015	All n = 2185
	(column percent; *mean (SD) in italics*)			
**Age at baseline (years)**	*48.1(6.7)*	*49.9(6.5)*	*50.5(6.4)*	*50.4(6.4)*
**Age <50**	57.1	43.6	39.2	39.7
**Age >50**	42.9	56.4	60.8	60.3
**BMI (kg/m2)**	*25.1(5.8)*	*24.7(4.4)*	*24.2(4.1)*	*24.3(4.1)*
**BMI <20**	9.5	7.4	10.3	10.1
**BMI ≥20 < 25**	52.4	51.7	55.0	54.7
**BMI ≥25 < 30**	23.8	29.5	25.2	25.5
**BMI ≥30**	14.3	11.4	9.5	9.7
**Age at menarche**				
**<12 years**	9.5	14.1	12.6	12.6
**>12 years**	90.5	84.6	86.7	86.6
**Missing**	-	1.3	0.7	0.7
**Oral contraceptives**				
**Yes**	9.5	6.0	7.7	7.6
**No**	90.5	93.3	91.9	92.0
**Missing**	-	0.7	0.3	0.4
**Postmenopausal**				
**Yes**	47.6	56.4	61.0	60.5
**No**	52.6	43.6	39.0	39.5
**Children**				
**Yes**	76.2	77.9	83.1	82.6
**No**	23.8	21.5	16.7	17.1
**Missing**	-	0.7	0.2	0.3
**HRT in postmenopausal**				
**Yes**	10	11.9	15.7	15.4
**No**	90	88.1	84.2	84.5
**Missing**	-	-	0.1	0.1
**Smoking status**				
**Never**	42.9	51.7	46.3	46.6
**Ex**	23.8	20.1	19.0	19.1
**Current**	33.3	27.5	34.5	34.0
**Missing**	-	0.7	0.3	0.3
**Married**				
**Yes**	42.9	60.4	52.4	52.9
**No**	47.6	31.5	42.0	41.3
**Missing**	9.5	8.1	5.6	5.8
**Alcohol consumption**				
**None**	9.5	20.8	14.7	15.1
**Less than every week**	57.1	53.7	60.2	59.7
**Every week**	33.3	24.8	24.6	24.7
**Missing**	-	0.7	0.5	0.5
**Education**				
**≤12 years**	76.2	79.1	81.6	81.4
**>12 years**	23.8	16.9	14.4	14.6
**Missing**	-	4.0	4.0	4.0

*Including one case where information on CIS or invasive status was not known.

**Table 2:** **Distribution of potential risk factors for breast cancer according to serum T3 level**

Factor			T3 quartile	
	1	2	3	All
	n = 682	n = 765	n = 738	n = 2185
	Column percent (*T3 levels in italics*)			
T3 (mIU/l)	*<1.70*	*1.70-1.90*	*>1.90*	
Age				
< 50	60.6	37.9	22.4	39.7
> 50	39.4	62.1	77.6	60.3
Education				
<12 years	55.1	55.9	62.5	57.9
12 years	20.8	26.9	22.4	23.5
>12 years	19.6	13.5	11.2	14.6
Missing	4.4	3.7	3.9	4.0
Married	56.7	51.8	50.4	52.9
Missing	0.7	3.8	12.6	5.8
Smoking				
Never	44.0	48.5	47.0	46.6
Current	35.3	33.3	33.3	34.0
Ex	20.7	18.0	18.8	19.1
Missing	-	0.1	0.8	0.3
Alcohol consumption				
Nothing	13.5	14.0	17.8	15.1
Less than every week	56.9	60.9	61.0	59.7
Every week	29.5	25.0	20.1	24.7
Missing	0.1	0.1	1.2	0.5
Menarche < 12 years	14.4	12.7	11.0	12.6
Missing	0.1	0.7	1.4	0.7
OC-use	4.5	7.1	11.1	7.6
Missing	-	0.1	0.9	0.4
Nulliparity	17.4	15.4	18.6	17.1
Missing	-	0.1	0.7	0.3
Peri/postmenopausal	41.2	63.3	75.5	60.5
HRT in peri/postmenopausal	13.9	13.8	17.6	15.4
Missing	-	-	0.2	0.1
Height				
≤160	24.6	30.6	33.3	29.7
>160 ≤ 165	32.7	32.3	31.0	32.0
>165 ≤ 170	27.0	22.9	24.3	24.6
>170	15.7	14.2	11.4	13.7
BMI				
<20	13.8	8.5	8.3	10.1
≥20 < 25	62.2	55.0	47.6	54.7
≥25 < 30	18.3	27.7	29.8	25.5
≥30	5.7	8.8	14.4	9.7

### All breast tumours

The third T3 tertile had a statistically significant association with “all” breast cancers in the analysis adjusted for age and OC (Table 3). This was also confirmed in the continuous analysis (Table 4), and the association was even stronger in postmenopausal women (Table 5). All of the results regarding “all” tumours, and also regarding all other analyses below, were similar when cases diagnosed during the first two years following baseline were excluded (data not shown).

**Table 3:** **T3 tertiles and risk of specific prognostic factors in breast cancer**

Tumour group	Tertile*	Cases	HR	HR**	HR***
		(n)	(95% CI)	(95% CI)	(95% CI)
**All**	**Tertile 1**	38	1.0	1.0	1.0
**n = 149**	**Tertile 2**	54	1.25 (0.85-1.84)	1.33 (0.90-1.97)	1.38 (0.93-2.04)
	**Tertile 3**	57	1.34 (0.92-1.97)	1.50 (1.01-2.23)	1.61 (1.07-2.43)
**Grade I**	**Tertile 1**	12	1.00	1.00	1.00
**n = 42**	**Tertile 2**	20	1.51 (0.74-3.09)	1.57 (0.76-3.26)	1.68 (0.81-3.49)
	**Tertile 3**	10	0.79 (0.34-1.82)	0.84 (0.35-2.01)	0.97 (0.40-2.37)
**Grade II**	**Tertile 1**	14	1.00	1.00	1.00
**n = 56**	**Tertile 2**	14	0.91 (0.43-1.19)	0.95 (0.45-2.01)	0.98 (0.46-2.69)
	**Tertile 3**	28	1.89 (1.00-3.50)	2.04 (1.04-4.00)	2.13 (1.06-4.30)
**Grade III**	**Tertile 1**	7	1.00	1.00	1.00
**n = 38**	**Tertile 2**	17	2.21 (0.92-5.32)	2.30 (0.94-5.61)	2.34 (0.96-5.74)
	**Tertile 3**	14	1.90 (0.77-4.71)	2.04 (0.80-5.23)	2.14 (0.82-5.59)
**Size ≤20 mm**	**Tertile 1**	28	1.00	1.00	1.00
**n = 93**	**Tertile 2**	31	1.01 (0.60-1.68)	1.04 (0.62-1.75)	1.09 (0.65-1.84)
	**Tertile 3**	34	1.15 (0.70-1.89)	1.21 (0.72-2.05)	1.36 (0.79-2.33)
**Size >20 mm**	**Tertile 1**	6	1.00	1.00	1.00
**n = 45**	**Tertile 2**	20	3.02 (1.21-7.53)	3.21 (1.28-8.07)	3.19 (1.26-8.06)
	**Tertile 3**	19	3.01 (1.20-7.53)	3.33 (1.30-8.58)	3.17 (1.20-8.36)
**Node positive**	**Tertile 1**	5	1.00	1.00	1.00
**n = 39**	**Tertile 2**	16	2.90 (1.06-7.92)	3.22 (1.17-8.87)	3.36 (1.22-9.30)
	**Tertile 3**	18	3.40 (1.26-9.16)	4.08 (1.47-11.32)	4.53 (1.60-12.83)
**Node negative**	**Tertile 1**	27	1.00	1.00	1.00
**n = 91**	**Tertile 2**	33	1.11 (0.67-1.85)	1.13 (0.67-1.89)	1.18 (0.70-1.99)
	**Tertile 3**	31	1.08 (0.65-1.82)	1.12 (0.65-1.92)	1.20 (0.69-2.10)
**ER positive**	**Tertile 1**	28	1.00	1.00	1.00
**n = 105**	**Tertile 2**	41	1.32 (0.82-2.15)	1.37 (0.84-2.23)	1.42 (0.87-2.32)
	**Tertile 3**	36	1.22 (0.74-1.99)	1.28 (0.77-2.15)	1.39 (0.82-2.36)
**ER negative**	**Tertile 1**	6	1.00	1.00	1.00
**n = 36**	**Tertile 2**	11	1.67 (0.62-4.52)	1.75 (0.64-4.77)	1.85 (0.68-5.09)
	**Tertile 3**	19	3.01 (1.20-7.53)	3.24 (1.25-8.40)	3.52 (1.32-9.41)
**PGR positive**	**Tertile 1**	27	1.00	1.00	1.00
**n = 97**	**Tertile 2**	36	1.21 (0.73-1.99)	1.25 (0.75-2.08)	1.27 (0.77-2.13)
	**Tertile 3**	34	1.19 (0.72-1.97)	1.26 (0.74-2.14)	1.32 (0.77-2.27)
**PGR negative**	**Tertile 1**	7	1.00	1.00	1.00
**n = 44**	**Tertile 2**	16	2.09 (0.86-5.07)	2.15 (0.87-5.29)	2.35 (0.96-5.80)
	**Tertile 3**	21	2.87 (1.22-6.75)	3.03 (1.25-7.34)	3.52 (1.42-8.75)

*Cut-offs: tertile 1 (<1.70 ), tertile 2 (1.70-1.90 ), and tertile 3 (>1.9 ), **adjusted for age, ***adjusted for age and oral contraceptives.

**Table 4:** **Continuous T3 and risk of specific prognostic factors in breast cancer**

Tumour group	HR (95% CI)	HR*(95% CI)	HR**(95% CI)
**All**	1.57 (1.06-2.32)	1.75 (1.18-2.60)	1.89 (1.27-2.82)
**Grade I**	0.93 (0.40-2.13)	1.00 (0.42-2.39)	1.20 (0.50-2.89)
**Grade II**	2.45 (1.32-4.55)	2.61 (1.40-4.87)	2.68 (1.42-5.06)
**Grade III**	1.68 (0.75-3.80)	1.78 (0.80-4.10)	1.86 (0.80-4.36)
**Size ≤20 mm**	1.53 (0.90-2.60)	1.65 (0.96-2.84)	1.88 (1.09-3.23)
**Size >20 mm**	1.98 (0.96-4.09)	2.11 (1.01-4.41)	1.96 (0.90-4.26)
**Node positive**	2.81 (1.39-5.67)	3.13 (1.57-6.23)	3.34 (1.70-6.56)
**Node negative**	1.18 (0.68-2.05)	1.22 (0.68-2.17)	1.32 (0.73-2.38)
**ER positive**	1.38 (0.83-2.28)	1.46 (0.87-2.46)	1.60 (0.95-2.71)
**ER negative**	3.95 (1.97-7.90)	4.15 (2.08-8.30)	4.32 (2.16-8.61)
**PGR positive**	1.38 (0.82-2.33)	1.48 (0.86-2.53)	1.57 (0.91-2.71)
**PGR negative**	3.38 (1.74-6.56)	3.53 (1.80-6.89)	3.89 (2.02-7.50)

RR *adjusted for age, RR **adjusted for age and oral contraceptives.

**Table 5:** **Continuous T3 and risk of specific prognostic factors in breast cancer in postmenopausal women**

Tumour group	RR (95% CI)	RR* (95% CI)	RR** (95% CI)
**All**	2.87 (1.86-4.43)	2.85 (1.86–4.37)	2.88 (1.90-4.37)
**Grade I**	2.32 (0.85-6.36)	2.35 (0.82-6.75)	2.37 (0.83-6.77)
**Grade II**	4.12 (2.19-7.77)	3.99 (2.17-7.36)	3.98 (2.19-7.23)
**Grade III**	1.18 (0.39-3.55)	1.24 (0.42-3.67)	1.30 (0.45-3.81)
**Size ≤20 mm**	3.28 (1.86-5.76)	3.31 (1.86-5.89)	3.33 (1.89-5.86)
**Size >20 mm**	1.64 (0.63-4.26)	1.76 (0.71-4.33)	1.83 (0.76-4.42)
**Node positive**	3.44 (1.54-7.66)	3.35 (1.59-7.07)	3.37 (1.63-6.96)
**Node negative**	2.08 (1.05-4.10)	2.08 (1.05-4.10)	2.12 (1.08-4.15)
**ER positive**	2.44 (1.38-4.32)	2.44 (1.39-4.29)	2.48 (1.43-4.31)
**ER negative**	4.83 (2.18-10.69)	4.72 (2.16-10.29)	4.69 (2.19-10.06)
**PGR positive**	2.59 (1.44-4.65)	2.59 (1.45-4.63)	2.62 (1.48-4.63)
**PGR negative**	3.87 (1.79-8.33)	3.79 (1.81-7.96)	3.81 (1.85-7.84)

RR *adjusted for age, RR **adjusted for age and oral contraceptives.

### Grade

Concerning ***NHG I*** tumours, there was no statistically significant association with T3 in the tertile or continuous analysis (Tables 3 and 4). Likewise, no association was seen after stratification for menopause status (Table 5). There was a statistically significant association between the highest T3 tertile and ***NHG II*** tumours. However in this subgroup the amount of cases was higher rendering higher statistical power and this alone could explain the significance concerning *NHG II*. This association was even stronger when adjusted for age and age and OC (Table 3). The association was also confirmed by the continuous analysis (Table 4), and even stronger among postmenopausal women (Table 5). There was no statistically significant association in any of the analyses concerning ***NHG III*** tumours.

### Tumours size

There was no association between T3 and tumours with a ***size ≤ 20 mm*** in the tertile analysis (Table 3). However, in the adjusted continuous analysis, there was a statistically significant positive association (Table 4) which was even more pronounced in postmenopausal women (Table 5). Positive, statistically significant associations were found in the T3 tertile analyses, for tumours with a ***size > 20 mm*** (Table 3). This association was weaker in the continuous analysis and statistically significant only in the analysis adjusted for age (Table 4). After stratification for menopausal status the association was lost (Table 5).

To summarize, the tertile analysis showed a high risk for large tumours ( >20 mm), but not for small tumours. The continuous analysis was only statistically significant for small tumours (even if the point estimate for large tumours was actually slightly higher). In postmenopausal women, there was still a high risk of large tumours (HR: 1.83), but it was not statistically significant and the risk for small tumours was clearly higher (HR: 3.33).

### Nodal status

There was a statistically significant positive association in a dose response pattern between T3 tertiles and the risk of a ***positive nodal status*** (Table 3). The association grew stronger when adjusted and was confirmed by the continuous analysis (Table 4) and in the analysis including only postmenopausal women (Table 5). Concerning ***negative nodal status***, there was no association with T3 levels in the tertile or continuous analyses (Tables 3 and 4), but in the postmenopausal women, there was a positive statistically significant association that grew stronger after adjustment (Table 5). Comparing the risk of different nodal status in postmenopausal women, the risk was higher for node positive tumours (HR: 3.37) as compared to node negative tumours (HR: 2.12).

### ER status

There was no statistically significant association in the T3 tertile and continuous analysis concerning tumours with ***positive ER status*** (Tables 3 and 4). However, in postmenopausal women, the association was statistically significant both in the crude and adjusted analysis (Table 5). T3 levels in the highest tertile were associated with a high risk of tumours with a ***negative ER status*** (Table 3). These statistically significant associations remained in the continuous analysis and the analysis restricted to menopausal women (Tables 4 and 5). Among postmenopausal women, the association with ER negative tumours (HR: 4.69) was clearly higher than the association with ER positive tumours (2.48).

### PGR status

There was no statistically significant association in the tertile and continuous analysis and ***positive PGR status*** (Tables 3 and 4). There was however, a positive statistically significant association among postmenopausal women (Table 5). Concerning ***negative PGR status***, there was a statistically significant association in the highest tertile (Table 3) and the association remained in the continuous models (Tables 4 and 5). The highest HR in postmenopausal women was seen in relation with PGR negative tumours.

## Discussion

This is the first prospective study on serum T3 levels in relation to prognostic factors in breast cancer. It shows a positive association between higher serum concentrations of T3 and presence of lymph node metastases and negative ER and PGR status. The association of T3 with these negative prognostic factors is also interesting in comparison with our previous study [11], where there was an association of T3 with an increase in mortality from breast cancer. Although not fully comparable, these findings indicate a possible common component.

This study was based on a cohort of middle-aged women with a high participation rate (70%) [12], that is 30% did not take part therefore absolute risks may not be applicable to all age groups or to the general population. Due to a wide distribution of T3 levels, internal comparisons were made and our estimates of relative risks should not be affected by selection bias.

The Swedish Cancer Registry and the Swedish Cause-of-Death Registry have been validated and found to have a completeness of about 99% [18], resulting in a very complete follow up.

It is unlikely that women with T3 levels within the upper limit of the normal range are more prone to participate in mammography screening than others, hence a possible detection bias in hyperthyroid breast cancer patients was probably not present.

There is a well-known circadian variation concerning T3 [19]. Ranking of individuals over time is, however, quite stable [20]. A true variation over time would most likely have led to an un-differential misclassification of T3 and, hence, attenuated observed risks.

The use of total T3 as a marker of T3 status involves an important methodological aspect. Most T3 in the circulation is bound to three transport proteins, thyroxin binding globulin (TBG), transthyretin and albumin. An increased TBG concentration leads to higher levels of total T3, and this is important to consider in studies of breast cancer risk, as exogenous oestrogens, that are risk factors for breast cancer, lead to increased TBG binding capacity [21]. This was handled by a sensitivity analysis were women treated with HRT and OC were excluded, and by adjusting for these confounders in all the analyses. The results after exclusion were very similar.

A limitation of the study is that in the baseline questionnaire used to collect data from the participants, there were no questions on co-morbidities or medication which may affect thyroid function. Neither was there information on thyroxin use specifically. This was handled by excluding women who affirmed any treatment for *goitre*, which probably limited the interference of unreported thyroxin use with the results in the present study.

The current study is the first prospective study to date on T3 levels and prognostic factors in breast cancer and therefore there is no expected strength of the associations. Hence, we have not performed a priori power calculation. In our study, the number of cases was quite limited leading to wide confidence intervals and low statistical precision. This led to an imprecision in the estimates related to T3, and the lack of association between T3 and breast cancer histological grade III could be due to a type II error. Furthermore, statistical precision was an even more serious problem in analyses stratified for menopausal status, and these results, with very wide CI:s, should be regarded with caution.

An additional problem related to the limited number of breast cancer cases in the different subgroups was that not all confounders could be included at the same time in the final model for each respective endpoint. However, only the factor included, namely OC, had a substantial change off effect, 0.14. This indicates that the lack of adjustment for all factors did not seriously confound the observed HRs.

The potential association between thyroid hormones and outcome following breast cancer has been discussed for more than a century. Studies on the relation to breast cancer aggressiveness have however been very scarce. We found no prospective study on the association of T3 levels and prognostic factors in breast cancer.

In a cross-sectional study, it has been shown that thyroid autoimmunity in relation to breast cancer aggressiveness is associated with a lower frequency of distant metastasis [6]. Another cross-sectional study on thyroid conditions and breast cancer aggressiveness found no relation to the histopathological grade but higher frequency of metastatic lymph nodes and vascular invasion in patients with thyroid pathology [7]. This finding was in line with a recent experimental study that showed an increased invasiveness and formation of metastasis in breast cancer of hypothyroid mice [22]. On the contrary, an early cross-sectional study by Lemaire et al., showed no relation between thyroid function and TNM stage of breast cancer [8]. A problem in the interpretation of the above studies is that thyroid hormones may indeed be affected by prevalent breast cancer, stress, or treatment. Breast cancer *per se*, as well as its treatment, may affect hormonal levels. Such a condition, confounding the results of cross sectional studies is the non-thyroidal illness syndrome. This is a condition characterised by abnormal thyroid function test results in patients with acute or chronic systemic illnesses without underlying thyroid disease. The laboratory parameters of this syndrome include low serum levels of T3, with normal or low levels of T4 and normal or low levels of TSH [23]. We do not believe that non-thyroidal illness syndrome was an obstacle in our study since women with prevalent breast cancer and cancer diagnosed up to two years following baseline were excluded. Furthermore, for the baseline examination, the women attended the hospital from their homes without any assistance, it is therefore unlikely that they were suffering from a serious underlying disease when the samples were taken.

We found that T3 levels were positively associated with invasive breast cancer in general, both in the tertile and continuous analyses. As previously reported [9], this association was also present after stratification for menopause status. Interestingly, these findings are in line with experimental studies that have shown that thyroid hormones influence both normal breast cell differentiation [24] and breast cancer cell proliferation [25–28].

Nottingham grade is an independent determinant of survival in breast cancer. When using NHG as the endpoint we found a statistically significant association with T3 only in grade II. If this merely reflects a higher amount of cases – and therefore a higher statistical power in this group – or if it depends on certain tumour characteristics being more common in grade II tumours remains an open question at present.

There was a positive association between the third T3 tertile and large tumours (>20 mm), but not for small tumours. The continuous analysis showed a similar, high, risk for both small and large tumours, although only statistically significant for small tumours. In postmenopausal women, there was a high risk of large tumours, but it was not statistically significant and the risk for small tumours was clearly higher. Taken together these findings suggest that there may be an association between high thyroid hormone levels and large tumours, but the even stronger association with small tumours in postmenopausal women is intriguing and together with the fact that CIs were generally wide, these findings need to be confirmed. Given the overall association with large sized tumours this could be explained by the proliferative effect of T3 on breast cancer cell lines reported in experimental studies [24–27], but further studies specifically on tumour size would be of value.

All analyses showed a positive association with presence of lymph node metastases. In postmenopausal women there was also a statistically significant association with negative lymph node status. To clarify this finding, a heterogeneity analysis was performed and showed to be statistically significant for the point estimate in positive lymph node status.

In the present study, there was an overall positive association between serum T3 levels and ER *negative* breast cancer. It has been shown that T3 binds and stimulate ERs, acting in synergy with oestrogen on breast cancer cell lines, potentiating the oestrogenic effect and enhancing cell proliferation [28]. Our finding implicates that T3 may also affect breast cancer by some other mechanism(s) in addition to ER stimulation. An interesting possibility involves the thyroid hormone receptors (TR) α and β and their isoforms, which have been demonstrated by various methods in breast cancer cells [29, 30]. Another observation of interest in this context involves the HER2 gene, which is of fundamental importance in the current evaluation and treatment of breast cancer. This gene is located close to the gene for TRα on the long arm of chromosome 17, and amplification of the HER2 gene is often accompanied by co-amplification of the gene for TRα [31]. In addition, point mutations in breast cancer, that may affect tumour behaviour, have also been detected in the genes encoding TRα and TRβ [32].

Further investigations in this field are called for, not least considering the potential clinical implications for the care of women with disorders or treatments resulting in increased serum concentrations of T3. Judging from recent observations in various types of malignant tumour disease, this line of research may also lead to new strategies for treatment of breast cancer [32, 33].

## Conclusions

This prospective study of serum T3 levels in relation to breast cancer aggressiveness is to our knowledge, the first of its kind. We found statistically significant positive associations between higher prediagnostic T3 levels and occurrence of lymph node metastases, and negative ER and PGR status. Furthermore, the association between higher T3 levels and increased mortality in breast cancer, as demonstrated by us previously, can be related to both a higher incidence and more aggressive forms of breast cancer.

## Abbreviations

T3: Triiodothyronine
HR: Hazard ratio
ER: Oestrogen receptor
PGR: Progesterone receptor
OC: Oral contraceptives
BMI: Body mass index
TSH: Thyroid stimulating hormonr
RIA: Radioimmunoassay
NHG: Nottingham grade
IHC: Immunohistochemical
LB: Lennart bondeson
CI: Confidence interval
TBG: Thyroid binding globulin
HRT: Hormone replacement therapy
TR: Thyroid receptor.
